# Predictive Value of Neutrophil to High-Density Lipoprotein Ratio for Contrast-Induced Acute Kidney Injury for Patients with Acute Myocardial Infarction Undergoing Primary Percutaneous Coronary Intervention

**DOI:** 10.31083/j.rcm2402059

**Published:** 2023-02-10

**Authors:** Zhen Wang, Yanan Li, Guoqi Shen, Hang Qiu, Yinghua Zhu, Di Zheng, Wenhua Li

**Affiliations:** ^1^Institute of Cardiovascular Diseases, Xuzhou Medical University, 221000 Xuzhou, Jiangsu, China; ^2^Department of Cardiology, Affiliated Hospital of Xuzhou Medical University, 221000 Xuzhou, Jiangsu, China

**Keywords:** acute myocardial infarction, percutaneous coronary intervention, contrast-induced acute kidney injury, neutrophil to high-density lipoprotein ratio, neutrophil to lymphocyte ratio

## Abstract

**Background:**

To investigate the incidence of contrast-induced acute kidney injury (CI-AKI) in patients with acute myocardial infarction (AMI) undergoing primary percutaneous coronary intervention (PCI) in relation to the neutrophil to high-density lipoprotein cholesterol ratio (NHR), and to further compare the predictive value of NHR and the neutrophil to lymphocyte ratio (NLR) for CI-AKI.

**Methods:**

We retrospectively analyzed 1243 AMI patients undergoing PCI from January 2019 to December 2021, and collected creatinine within 72 h after PCI. All patients were divided into a CI-AKI group and non-CI-AKI group according to the definition of CI-AKI, and the clinical information of the two groups was compared. Potential risk factors for CI-AKI in AMI patients undergoing primary PCI were screened by using logistic regression analysis, and receiver operating characteristic (ROC) curves were used to compare the predictive value of NHR and NLR.

**Results:**

A high NHR and high NLR were correlated with a high incidence of CI-AKI in AMI patients undergoing primary PCI, and NHR (odds ratio (OR): 1.313, 95% confidence interval (CI): 1.199–1.438) and NLR (OR: 1.105, 95% CI: 1.041–1.174) were independent risk factors for CI-AKI (*p *< 0.05). Compared with NLR, the area under the curve (AUC) of NHR was larger (AUC = 0.668, 95% CI: 0.641–0.694 vs. AUC = 0.723, 95% CI: 0.697–0.748), and the difference was significant (*p *< 0.05), with higher sensitivity (61.67% vs. 70.83%) and specificity (64.91% vs. 66.10%).

**Conclusions:**

Compared with the NLR, the NHR is more valuable in predicting the incidence of CI-AKI in AMI patients undergoing primary PCI.

## 1. Introduction

Cardiovascular interventions have become an important method for the clinical diagnosis and treatment of cardiovascular diseases, and the number of adverse effects caused by contrast agents has also increased. Contrast-induced acute kidney injury (CI-AKI) is the third leading cause of hospital-acquired renal failure, after renal artery hypoperfusion and nephrotoxicity of drugs [1]. The mechanism of CI-AKI is complex and unclear. Its incidence may be related to underlying renal disease, renal artery hypoperfusion, and the toxic effects of contrast agents on renal tubules, which lead to tubular obstruction, renal medullary hypoxia, oxygen-free radical damage, apoptosis, and immune and inflammatory responses [2]. CI-AKI is associated with longer hospital stays and higher health care costs, and has become an important disease affecting the health of the population. However, no effective treatment is available, so the early identification of CI-AKI is critical.

Neutrophils are the predominant leukocyte type in acute inflammation and are mediators of the early inflammatory response. Neutrophils not only release cytotoxic substances but also promote the release of reactive oxygen species, leading to local ischemia, plaque instability, and thrombosis [3]. Several studies have confirmed the inflammatory response as a risk factor for CI-AKI [4, 5]. High-density lipoprotein (HDL) has a strong anti-atherosclerotic function. In healthy populations, HDL also has anti-inflammatory and antioxidant abilities, promotes endothelial repair, and acts as a systemic signal. HDL was reported to correlate with CI-AKI [6]. HDL can regulate activated neutrophils. In contrast, the structure and content of HDL can be altered by activated neutrophils [7]. Therefore, we investigated whether the neutrophil to high-density lipoprotein ratio (NHR) is a novel indicator of inflammation and lipid levels, and predicts the development of CI-AKI. In addition, inflammation-based scores have been widely used to predict the incidence of CI-AKI in recent years, and the neutrophil to lymphocyte ratio (NLR) is an independent risk factor for CI-AKI [8].

Therefore, we further compared the predictive value of NLR and NHR for CI-AKI in patients with AMI undergoing primary percutaneous coronary intervention (PCI).

## 2. Methods

### 2.1 Subjects

Between January 2019 and December 2021, 1243 patients with AMI who underwent primary PCI at Xuzhou Medical University Hospital (Jiangsu Province, China) were retrospectively and consecutively enrolled in present study. The primary outcome of this study was the incidence of CI-AKI. AMI includes ST-segment elevation myocardial infarction (STEMI) and non-ST-segment elevation MI (NSTEMI). AMI [9] was defined as: the presence of (1) typical chest pain and/or ischemic symptoms at rest lasting >20 min; (2) ST-segment elevation consistent with MI ≥2 mm in adjacent chest leads and/or ST-segment elevation ≥1 mm in ≥2 standard leads, new (or presumably new) left bundle branch block on admission electrocardiogram, ischemic T inversion or >0.5 mm ST segment depression on ≥2 consecutive leads; and (3) positive markers for myocardial necrosis (cardiac troponin (cTn) or high sensitivity cTn (hs-cTn) >upper limit of normal or doubling of hs-cTn within 3 h). Diagnostic criteria for CI-AKI depend on the European Society of Urogenital Radiology [10]: an increase in serum creatinine level of 0.5 mg/dL (44.2 μmol/L) or 25% from baseline between 48 and 72 h after contrast agents administration, excluding other causes of kidney injury. Exclusion criteria included patients with incomplete basic information, receiving hemodialysis or estimated glomerular filtration rate (eGFR) <15 mL/(min×1.73 $m^{2}$), an autoimmune disease, recent (past 3 days) use of contrast agents, recent (within 72 h before and 72 h after surgery) use of potentially nephrotoxic drugs, malignancy, or death. The study protocol was approved by the ethics committee of the Affiliated Hospital of Xuzhou Medical University (Protocol No. XYFY2022-KL122-01).

### 2.2 PCI Procedure and Medications

PCI was performed by an interventional cardiologist using a radial or femoral artery approach according to standard clinical practice. All patients were given aspirin (loading dose, 300 mg), clopidogrel (loading dose, 300 mg), or ticagrelor (180 mg) at the time of presentation, followed by aspirin (100 mg/day), clopidogrel (75 mg/day), or ticagrelor (180 mg/day). The contrast agent used was a low-osmolar nonionic contrast agent with an osmotic concentration of 600–800 mOsm/Kg. After the procedure, depending on the patient’s underlying physical condition, the patient was given an appropriate amount of fluid hydration by an interventional cardiologist to facilitate metabolism of the contrast agent in the body.

### 2.3 Laboratory Parameters

Blood samples were collected from the anterior elbow vein prior to PCI, and the blood samples were tested in our central laboratory, analyzed by the biochemistry laboratory, and reported uniformly. The XE-5000 automatic hematology analyzer (Sysmex Co., Kobe, Japan) was used for blood cell analysis. The HLC-723G8 analyzer (Tosoh, Tokyo, Japan) was used to detect glycated hemoglobin, and the detection reagents were from Tosoh (Shanghai, China) Biotechnology. The Cobas 8000 automatic biochemical analyzer (Roche, Mannheim, Germany) was used to detect the serum levels of cholesterol, triglyceride, prealbumin, albumin, total bilirubin, direct bilirubin, fasting blood glucose, uric acid, urea, creatinine, HDL, and LDL (detection reagents were from Shanghai Deacon Company, Shanghai, China). The eGFR was calculated using a simplified Modification of Diet in Renal Disease formula: eGFR (mL/min/1.73 $m^{2}$) = 186 × serum creatinine ${\mathrm{(mg/dL)}}^{-1.154}$ × ${\mathrm{age}}^{-0.203}$ × (0.79 female).

### 2.4 Statistical Analyses

The Shapiro-Wilk test was used to characterize the distribution of the data. The mean ± standard deviation was used to represent the measurement data, the median to the count data, and the ratio or composition ratio to the categorical data. The *t*-test was used for normally distributed data, U-test for non-normally distributed data, and χ^2^ test for count data. Screening for potential risk factors for CI-AKI using logistic regression analysis. Variables that were significantly associated with CI-AKI (*p <* 0.05) but not included in the NLR and NHR were separately entered into a multivariate model. The Hosmer-Lemeshow statistic was used to assess the fit of the multivariate regression model. Statistical analyses were performed using SPSS version 26.0 (SPSS Inc., Chicago, IL, USA) and MedCalc version 11.4.2 (MedCalc Software, Mariakerke, Belgium). *p <* 0.05 was considered statistically significant.

## 3. Results

### 3.1 Baseline Characteristics

A total of 1243 AMI patients underwent primary PCI. The mean age of the patients was 63 ± 12 years; 316 were female (25.4%) and 240 patients (19.3%) developed CI-AKI after PCI. In total, 583 of the 1243 AMI patients had NSTEMI and 98 developed CI-AKI (16.8%). CI-AKI developed in 142 of 660 patients (21.5%) with STEMI. The incidence of CI-AKI was higher in STEMI than NSTEMI patients.

### 3.2 Comparison of Baseline Information of CI-AKI and Non-CI-AKI

Compared with patients without CI-AKI, patients who developed CI-AKI were older, predominantly female, and had a lower left ventricular ejection fraction (*p <* 0.05). However, there was no statistically significant difference between the CI-AKI and non-CI-AKI groups in terms of patients with hypertension, diabetes mellitus, and contrast dosage (*p >* 0.05). By analyzing the patients’ preoperative laboratory parameters, we found that neutrophils, NLR, N-terminal natriuretic peptide precursor (NT-proBNP), creatine kinase isoenzyme (CK-MB), NHR, and fasting glucose were higher in the CI-AKI group than in the non-CI-AKI group; whereas lymphocytes, triglycerides (TG) and HDL were lower, and the differences were statistically significant (*p <* 0.05). By observing the patients’ medication use, we found a statistically significant difference in the number of diuretics used in the CI-AKI and non-CI-AKI groups (*p <* 0.05; Table 1).

**Table 1.** **Comparison of general information between the CI-AKI and non-CI-AKI groups**.

Projects		CI-AKI (n = 240)	non-CI-AKI (n = 1003)	*p*
Basic information				
	Age, years	65.49 ± 11.65	62.76 ± 12.84	0.003
	Sex, female, n (%)	87 (36.3%)	229 (22.8%)	<0.001
	High blood pressure, n (%)	117 (48.8%)	462 (46.1%)	0.453
	Diabetes, n (%)	71 (29.6%)	259 (25.8%)	0.236
	Left ventricular ejection fraction (%)	51.35 ± 7.22	53.31 ± 7.64	0.002
	Contrast agent >100 mL (%)	147 (61.3%)	645 (64.3%)	0.194
Laboratory metrics				
	White blood cell count (×${\mathrm{10}}^{9}$/L)	9.83 ± 3.17	9.42 ± 3.05	0.064
	Lymphocyte count (×${\mathrm{10}}^{9}$/L)	1.39 ± 0.94	1.70 ± 1.13	<0.001
	Neutrophil count (×${\mathrm{10}}^{9}$/L)	8.43 ± 3.18	6.91 ± 3.59	<0.001
	NLR	8.25 ± 6.47	5.77 ± 6.07	<0.001
	CRP (mg/L)	14.22 ± 30.42	14.08 ± 31.19	0.950
	Red blood cell count (×${\mathrm{10}}^{9}$/L)	4.42 ± 0.63	4.43 ± 0.63	0.797
	Hemoglobin (g/L)	137.66 ± 16.80	138.98 ± 17.85	0.297
	Platelet count (×${\mathrm{10}}^{9}$/L)	202.92 ± 56.39	209.93 ± 61.43	0.107
	LN NT-proBNP	7.17 ± 1.15	6.78 ± 1.21	<0.001
	cTnT (ng/mL)	2.54 ± 1.23	2.02 ± 1.95	0.619
	CK-MB (m/L)	68.66 ± 64.70	53.84 ± 64.07	0.016
	Fibrinogen (g/L)	3.12 ± 1.23	3.03 ± 1.14	0.355
	AT3 (%)	83.38 ± 13.68	83.66 ± 11.78	0.809
	Albumin (g/L)	38.90 ± 3.50	38.90 ± 4.21	0.985
	TG (mmol/L)	1.42 ± 0.71	1.64 ± 1.42	0.021
	TC (mmol/L)	4.37 ± 1.00	4.43 ± 1.66	0.646
	LDL (mmol/L)	2.73 ± 0.84	2.74 ± 0.91	0.809
	HDL (mmol/L)	0.86 ± 0.25	1.00 ± 0.26	<0.001
	NHR	10.42 ± 4.36	7.31 ± 3.93	<0.001
	Serum creatinine (μmol/L)	68.88 ± 33.65	67.57 ± 51.16	0.628
	eGFR (mL/min)	111.33 ± 31.62	115.08 ± 36.04	0.109
	Fasting blood sugar (mmol/L)	7.03 ± 2.86	6.53 ± 2.68	0.013
	Glycation (%)	6.69 ± 1.43	6.61 ± 1.51	0.490
Drug administration				
	Aspirin, n (%)	240 (100%)	1001 (99.8%)	0.489
	Clopidogrel, n (%)	240 (100%)	1003 (100%)	-
	B-receptor blockers, n (%)	204 (85.0%)	836 (83.3%)	0.534
	ACEI/ARB, n (%)	150 (62.5%)	581 (57.9%)	0.177
	Statin, n (%)	236 (98.3%)	1003 (100%)	0.846
	CCB, n (%)	19 (7.9%)	94 (9.4%)	0.479
	Diuretics, n (%)	140 (58.3%)	350 (34.9%)	<0.001
	Nitrates, n (%)	112 (46.7%)	454 (45.3%)	0.695
	Low molecular heparin, n (%)	194 (80.8%)	762 (76.0%)	0.254

Values are presented as the mean ± SD, number (%) or median (interquartile range). NLR, neutrophil to lymphocyte ratio; CRP, C-reactive protein; NT-proBNP, N-terminal natriuretic peptide precursor; CK-MB, Creatine kinase isozyme; cTnT, cardiac troponin T; AT3, Thrombin III; TG, total triglycerides; TC, total cholesterol; HDL, high-density lipoprotein; LDL, low-density lipoprotein; NHR, neutrophil to high density lipoprotein ratio; eGFR, estimated glomerular filtration rate; ACEI, angiotensin-converting enzyme inhibitor; ARB, angiotensin II receptor blockers; CCB, calcium channel blocker.

### 3.3 Comparison of the Incidence of CI-AKI in Different Grades of NHR and NLR 

Based on the quartiles of NHR and NLR, all patients were divided into four groups: NHR, NLR ≥75%; NHR, NLR 75–50%; NHR, NLR 50–25%; and NHR, NLR <25%. The incidence of CI-AKI in each of the four groups was compared, as shown in Table 2. The incidence of CI-AKI in the NHR ≥75% group (35.7%) and NLR ≥75% group (30.9%) was higher than that in the other groups, with a significant difference between groups (*p <* 0.05).

**Table 2.** **Incidence of CI-AKI in different grades of NLR and NHR**.

Quartile	≥75%	75–50%	50–25%	<25%
NLR	96 (30.9%)	74 (23.8%)	43 (13.8%)	27 (8.7%)
NHR	120 (35.7%)	61 (21.4%)	39 (12.5%)	20 (6.5%)

NLR, neutrophil to lymphocyte ratio; NHR, neutrophil to high density lipoprotein ratio.

### 3.4 Multivariate Logistic Regression Analysis 

To assess the risk factors for CI-AKI, the influential factors (age, sex, left ventricular ejection fraction (LVEF), lymphocytes, neutrophils, NLR, Ln NT-proBNP, CK-MB, TG, HDL, NHR, fasting glucose, and diuretics) associated with CI-AKI and eGFR were subjected to univariate analysis. The results showed that age, sex, LVEF, lymphocytes, neutrophils, NLR, Ln NT-proBNP, CK-MB, TG, HDL, NHR, fasting glucose, and diuretics were all potential independent risk factors for CI-AKI. To exclude the confounding factors, the indicators (age, sex, LVEF, NLR, Ln NT-proBNP, CK-MB, TG, NHR, fasting glucose, and diuretics) not included in NLR and NHR were included in the multivariate analysis. To avoid interaction between NLR and NHR as both contained neutrophils, multivariate models were established separately (A and B), and the multivariate models were validated by Hosmer-Lemeshow goodness of fit (*p >* 0.05). The results showed that sex, NHR, and NLR were independent predictors of CI-AKI and were statistically significant (*p <* 0.05; Table 3). As shown in model A, the probability of CI-AKI was 4.257 times higher in female patients than in males; with each 1 increase in NHR, the probability of CI-AKI increased by 1.313 times. Similarly, in model B, the probability of CI-AKI was 3.686 times higher in female patients; with each 1 increase in NLR, the probability of CI-AKI in patients increased by 1.105 times.

**Table 3.** ** Multivariate logistic regression analysis of factors influencing CI-AKI in patients with AMI after PCI**.

Influencing factors	Univariate analysis			Multivariate analysis (Model A)			Multivariate analysis (Model B)		
	OR	95% CI	*p*	OR	95% CI	*p*	OR	95% CI	*p*
Age	1.018	1.006–1.030	0.003	1.020	0.991–1.049	0.177	0.997	0.971–1.024	0.849
Sex	1.922	1.421–2.599	<0.001	4.257	2.201–8.231	<0.001	3.686	1.972–6.887	<0.001
LVEF	0.968	0.948–0.989	0.003	0.984	0.940–1.031	0.503	0.969	0.927–1.012	0.157
Lymphocyte count	0.66	0.542–0.803	<0.001						
Neutrophil count	1.132	1.083–1.182	<0.001						
NLR	1.058	1.034–1.082	<0.001				1.105	1.041–1.174	0.001
LN NT-proBNP	1.338	1.171–1.529	<0.001	0.889	0.646–1.225	0.472	0.880	0.649–1.193	0.411
CK-MB	1.003	1.001–1.006	0.017	1.000	0.996–1.004	0.936	1.001	0.997–1.005	0.539
TG	0.814	0.687–0.965	0.018	0.808	0.577–1.132	0.216	1.075	0.821–1.409	0.598
HDL	0.083	0.042–0.162	<0.001						
NHR	1.192	1.149–1.236	<0.001	1.313	1.199–1.438	<0.001			
eGFR	1.003	0.999–1.008	0.110						
Fasting blood sugar	1.062	1.011–1.115	0.016	1.000	0.899–1.113	0.999	1.008	0.910–1.116	0.882
Diuretics	2.612	1.959–3.482	<0.001	1.475	0.773–2.817	0.239	1.670	0.898–3.105	0.105

CI, confidence interval; OR, odds ratio; LVEF, left ventricular ejection fraction; NLR, neutrophil to lymphocyte ratio; NT-proBNP, N-terminal natriuretic peptide precursor; CK-MB, Creatine kinase isozyme; TG, total triglycerides; HDL, high-density lipoprotein; NHR, neutrophil to high density lipoprotein ratio; eGFR, estimated glomerular filtration rate. Model A The variables included in multivariate analysis were the presence of age, sex, LVEF, Ln NT-proBNP, CK-MB, TG, NHR, fasting glucose, and diuretics. Model B The variables included in multivariate analysis were the presence of age, sex, LVEF, NLR, Ln NT-proBNP, CK-MB, TG, fasting glucose, and diuretics.

### 3.5 Receiver Operating Characteristic Curve Analysis

**Fig. 1.**
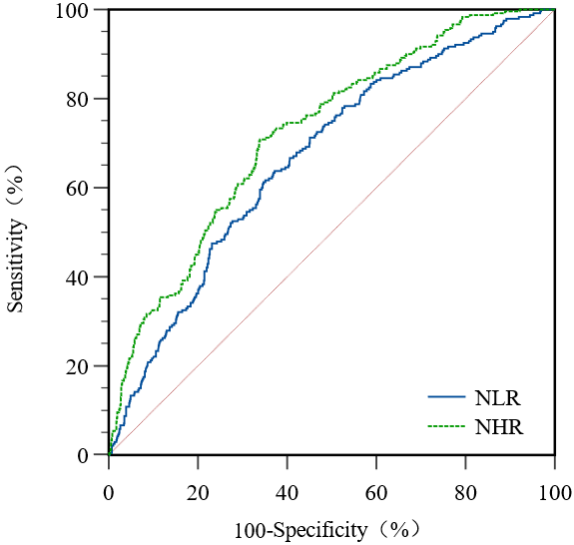
**ROC curves of patients with CI-AKI by NLR and NHR**. NLR, neutrophil to lymphocyte ratio; NHR, neutrophil to high density lipoprotein ratio; ROC, receiver operating characteristic.

Multivariate regression analysis showed that sex, NHR, and NLR were independent influencing factors of CI-AKI in AMI patients undergoing primary PCI. The receiver operating characteristic curves of NHR and NLR showed that the area under the curve of NHR was larger than that of NLR (AUC = 0.723, 95% CI: 0.697–0.748 vs. AUC = 0.668, 95% CI: 0.641–0.694), and the difference was significant (*p <* 0.05). The sensitivity (70.83%) and specificity (66.10%) of NHR were also better than that of NLR (sensitivity = 61.67%, specificity = 64.91%; Fig. 1, Table 4).

**Table 4.** **Comparison of ROC curves of NLR and NHR**.

	AUC	95% CI	*p*	Sensitivity	Specificity	Cut-off	Comparison of AUC	
							*p*	Z
NLR	0.668	0.641–0.694	<0.001	61.67%	64.91%	5.65	0.003	2.931
NHR	0.723	0.697–0.748	<0.001	70.83%	66.10%	7.64		

CI, confidence interval; AUC, area under curve; NLR, neutrophil to lymphocyte ratio; NHR, neutrophil to high density lipoprotein ratio; ROC, receiver operating characteristic.

## 4. Discussion

In the present study, we found that high preoperative NHR was strongly associated with the incidence of CI-AKI in AMI patients undergoing primary PCI and that NHR was an independent predictor of CI-AKI. Compared with NLR, NHR had better predictive value and better sensitivity and specificity. It can be concluded that NHR is a simple and easy inflammatory marker to predict the incidence of CI-AKI in AMI patients undergoing primary PCI.

In our study, we found that a higher proportion of STEMI patients who developed CI-AKI underwent PCI than NSTEMI patients, which might be due to the fact that STEMI is the result of transmural ischemia (that is, ischemia that involves the full thickness of the myocardium), whereas NSTEMI does not spread through all of the myocardial wall. Thus STEMI patients are hemodynamically unstable and more prone to hypotension or even shock, leading to inadequate renal perfusion and the development of CI-AKI [11]. We analyzed the data and found that the incidence of CI-AKI was higher in patients who used diuretics during treatment. The main effects of diuretics are to promote the excretion of sodium, chloride, and water, further reducing the effective blood volume and decreasing renal perfusion, leading to transient renal impairment and increasing the incidence of CI-AKI.

In recent years, a number of studies have reported the use of inflammatory factors for the assessment of prognosis in cardiac diseases, such as the systemic immune inflammation index, system inflammation response index, NLR, and NHR for the prediction of prognosis in transcatheter aortic valve implantation, off-pump coronary artery bypass, and PCI [12, 13]. CI-AKI is associated with increased morbidity and mortality, particularly in high-risk patients who have undergone PCI. The inflammatory response is an important risk factor for CI-AKI, and neutrophils are a systemic inflammatory marker that mediates the early inflammatory response. After the patient is exposed to the contrast agent, the contrast agent directly damages the kidney, followed by the infiltration of inflammatory cells such as macrophages, natural killer cells, lymphocytes, and especially neutrophils into the damaged tissue, leading to further destruction of the kidney [14]. Poppelaars *et al*. [15] found that C5aR2-deficient mice had reduced neutrophil activity, resulting in nephroprotective effects that led to lower creatinine levels and reduced acute tubular necrosis. Raup-Konsavage *et al*. [16] confirmed that peptidyl arginine deiminase-4 from neutrophils plays a pivotal role in renal ischemia/reperfusion-induced AKI. Núñez *et al*. [17] showed that lymphocytes are involved in the growth, development, rupture, and thrombosis of atherosclerotic plaques and that a decrease in lymphocyte count is associated with increased physiological stress, inflammatory response, and apoptosis in the organism. As in previous studies [18, 19], this study confirmed that neutrophils were increased and lymphocytes were decreased in AMI patients undergoing primary PCI who developed CI-AKI, and that neutrophils and lymphocytes are potential risk factors for CI-AKI.

HDL is a typical biomarker that responds to lipid metabolism and has a protective role in atherosclerotic and inflammatory processes. Its role is to transport excess cholesterol from peripheral tissues back to the liver for excretion [20]. In addition, HDL prevents the accumulation of monocytes into the arterial wall by inhibiting the expression of endothelial cell adhesion molecules. More importantly, HDL inhibits the activation, proliferation, and migration of neutrophils [21]. Cai *et al*. [22] showed that serum amyloid A leads to enhanced renal inflammation and elevated levels of urinary albumin and renal injury molecule-1, and significantly increases renal oxidative damage, which in turn damages the kidney, whereas HDL inhibits serum amyloid A and reduces the risk of renal injury. Park *et al*. [6] concluded that low HDL levels in people with normal renal function are at higher risk of chronic kidney disease (CKD), and that elevated HDL levels are associated with a reduced risk of CKD progression. It is recommended that more intensive measures to prevent CI-AKI be considered for patients with CKD with low HDL levels who are scheduled for PCI. Smith *et al*. [23] found that higher HDL before PCI treatment is associated with a lower incidence of CI-AKI, this view was also confirmed in our study.

The NLR is an effective predictor of cardiovascular risk in both primary and secondary prevention settings [24]. NLR as a mediator reflecting inflammation is being studied as a marker of CI-AKI. The NLR has predictive value not only for the incidence of CI-AKI in NSTEMI patients undergoing PCI [8], but also for STEMI patients as well [25]. Butt *et al*. [26] observed 1577 patients with AMI and concluded that elevated NLR is an independent predictor of CI-AKI in this patient population, which is consistent with the results of this study.

NHR is the ratio of neutrophils to HDL and combines the advantages of neutrophils and HDL. It is a potential novel biomarker for inflammation and lipids. Recent relevant studies have found that NHR can be used to predict retinal artery embolism [27], metabolic syndrome [28], acute ischemic stroke [29] and to assess the inflammatory process in Parkinson’s disease [30]. It is also widely used in cardiovascular diseases. A previous study by Kou *et al*. [21] showed that NHR was associated with the degree of coronary stenosis and can be used to predict severe coronary artery stenosis. Huang *et al*. [31] found that NHR may have predictive prognostic value for long-term mortality and recurrent MI by observing 528 elderly AMI patients (65–85 years), and was superior to the monocyte to HDL ratio and the LDL to HDL ratio. Li *et al*. [32] showed that NHR is a new independent risk factor for all-cause mortality in peritoneal dialysis patients and that NHR is correlated with kidney injury. Our study results show that NHR is an independent predictor of the incidence of CI-AKI in AMI patients undergoing primary PCI, and its predictive value is significantly better than NLR.

## 5. Limitations

The study had some limitations. First, this was a single-center retrospective observational study. Second, it was difficult to fully control for differences in baseline characteristics between groups. Finally, only preoperative NHR levels were recorded in this study, and postoperative NHR levels were not recorded and evaluated. Therefore, both the impact of preoperative high NHR on CI-AKI and whether treatment to reduce NHR will reduce the incidence of CI-AKI still require further evaluation in prospective randomized controlled trials with large samples.

## 6. Conclusions

NHR is not only an easily accessible marker but also an independent risk factor for the development of CI-AKI in patients with AMI undergoing primary PCI. Furthermore, NHR had better predictive value in detecting the incidence of CI-AKI compared with NLR. This helps clinicians to anticipate early and take timely preventive measures, thus reducing adverse events, reducing patients’ medical costs and improving their quality of life.

## Data Availability

The datasets generated and analyzed during the current study are not publicly available due to patient privacy, but are available from the corresponding author on reasonable request.
